# Bile Reflux Gastritis: Insights into Pathogenesis, Relevant Factors, Carcinomatous Risk, Diagnosis, and Management

**DOI:** 10.1155/2022/2642551

**Published:** 2022-09-12

**Authors:** Xiaolan Shi, Zijiao Chen, Yi Yang, Su Yan

**Affiliations:** Department of Gastroenterology, The First Affiliated Hospital of Soochow University, No. 899 Pinghai Road, Suzhou, 215006 Jiangsu Province, China

## Abstract

Bile reflux gastritis (BRG), a kind of gastrointestinal disorder in clinical practice, is characterized by regurgitation and inflammation. However, lack of guidelines leads to simple cognition and even ignorance of this disease for clinicians. Primarily, making the pathogenesis of BRG clear contributes to a correct and general understanding of this disease for physicians. Next, although recently there has been an increasing awareness among researchers in terms of the relevant factors for BRG, further studies involving large samples are still required to certify the relationship between them explicitly. Besides, researches have established that BRG is closely associated with the development of precancerous lesions and gastric cancer. Till now, there is still no golden standard for diagnosis of BRG. Nevertheless, advances in techniques, especially extensive applications of endoscopy and chemical analysis of reflux contents, have improved our ability to identify the occurrence of this disease as well as distinguishing physiological reflux from pathological reflux. Finally, it is fortunate for patients that more and more importance has been attached to the treatment of BRG. From lifestyle modification to drug therapy to surgery, all of them with the view of realizing symptomatic relief are employed for patients with BRG. In this review, we briefly evaluate this disorder based on the best available evidence, offering an overview of its complicated pathogenesis, diverse relevant factors, potential carcinomatous risk, modern diagnostic investigations, and effective therapeutic plans.

## 1. Introduction

Bile reflux gastritis (BRG) refers that owing to a variety of factors, the contents in the duodenum such as bile, pancreatic juice, and duodenal juice, which can damage the barrier of gastric mucosa, retrograde to stomach and lead to inflammation. In terms of its prevalence, a study including 804 cases where there was an endoscopic examination for abdominal pain found bile reflux was seen in 23.9% patients [1]. Another research observed that the prevalence of BRG was 16.7% and 61.8% in the control group, who had never undergone any biliary interventions and the postcholecystectomy group who had undergone cholecystectomy [2]. In addition, the presence of intragastric bile and varying degrees of inflammation can exist in the esophagus as well, which is defined as duodenogastroesophageal reflux (DGER). Basnayake et al. [3] demonstrated that DGER is increased in patients with gastroesophageal reflux disease (GERD). The prevalence of DGER among all GERD patients ranged from 10% to 97%.

In fact, bile reflux was first observed by William Beaumont in a patient with a gastrocutaneous fistula in 1833 [4]. However, the significance of this problem was not recognized until surgical operations that need resect or alter pylorus become usual. Afterward, BRG is increasingly discovered in individuals without gastric surgery, which is termed primary biliary reflux. Nowadays, as a kind of gastrointestinal disorders, BRG is deemed as a risk factor for gastric cancer but not taken seriously in gastroenterology department. Therefore, in this review, we aim to introduce its complicated pathogenesis, relevant risk factors, tough diagnosis, and particular treatment.

## 2. Pathogenesis

The occurrence of bile reflux gastritis includes two parts. One is reflux, the other is inflammation. Reflux takes place pathologically mainly due to gastroduodenal dysmotility, disorder of gastroduodenal hormones, or surgical operations. The prerequisite and foundation for it are reverse contractile activities of the duodenum and opening of the pyloric canal. Gastrointestinal hormones like gastrin, cholecystokinin, and secretin play an important role in reflux by influencing the secretion of gastric acids and regulating motility of the stomach [5]. Researches have established that the concentration of motilin (MTL) in patients with bile reflux shows little change and is absent for a peak compared with healthy individuals, which proves a connection between hormones and reflux [6]. With regard to surgery, it not only destroys the normal physiological structure of the stomach but also makes patients lose the inhibitory reflux effect of pyloric sphincter, which gives rise to duodenogastric reflux.

**Figure 1:**
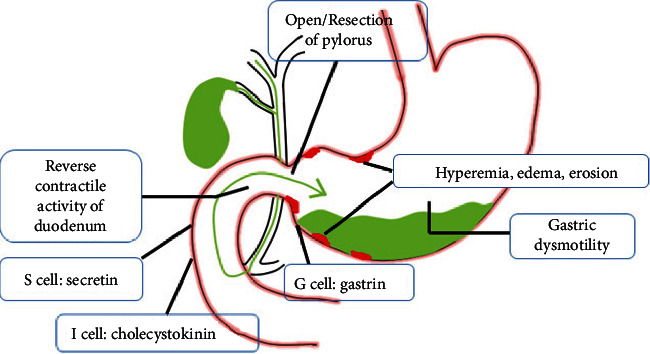
Pathogenesis of bile reflux gastritis. Reflux—gastroduodenal dysmotility, disorder of gastroduodenal hormones, and surgical resection of pylorus. Inflammation (hyperemia, edema, erosion)—stimulation of bile acids, lysolecithin, and so on.

Inflammation is primarily aroused by the stimulation of reflux contents. Among them, bile acids and lysolecithin are the major components that destroy the barrier on the surface of gastric mucosa by dissolving phospholipids and cholesterol, which motivates hydrogen ions in gastric juice to diffuse into gastric mucosa for the increased permeability of gastric epithelial cells [7–9]. As a consequence, the manifestations of gastritis such as hyperemia, edema, and erosion come up. The histological changes of the gastric mucosa perform foveolar hyperplasia and vascular congestion in the superficial layer. Besides, reflux liquids contain a mass of intestinal bacteria that can result in the imbalance of microbial flora in the stomach [10]. At the same time, the rise in the pH value due to alkaline bile creates a favorable environment for bacteria to breed, which aggravates the inflammation of gastric mucosa (Figure 1).

## 3. Relevant Factors

There are many factors associated with the development of bile reflux gastritis. Some of them are clarified to have a definite relationship with BRG while the others still remain controversial.

It is indicated that the rate of BRG is lower in middle-aged patients than in young and elderly ones [11]. Furthermore, males are less likely to get the disease of BRG compared with females [11]. In general, the morbidity of BRG is highest among young women.

In terms of smoking and drinking, they not only injure gastric mucosa but also loosen the sphincter of pylorus, which generate a reverse flow of bile to the stomach via slack pylorus [12]. Moreover, people who prefer sweet food or coarse food grains tend to have BRG. Sweet food can stimulate the secretion of gastric acids and hormones like glucagon and cholecystokinin (CCK) that inhibit motility and contraction of the stomach [13]. As for coarse food grains, the relaxation of gastric fundus slows gastric emptying, which weakens the capacity of the stomach to clear bile away. In addition, it is interesting to notice in clinical practice that individuals with BRG are often tall and slender. Gastroptosis that changes the normal physiological structure is suspected to play an important role in it.

A considerable proportion of diabetic patients suffer from gastroparesis, which is defined as a deficiency of gastric motility relevant to delayed gastric emptying. The principle of diabetic gastroparesis has been studied at home and abroad. It is described that long-term hyperglycemia can induce disorders of the autonomic nervous system, which reduce the tension of stomach and slow gastric peristalsis, thus leading to delayed gastric emptying and abnormal gastro-pyloric-duodenal dynamics that prolong residence time of bile in the stomach and contribute to the occurrence of duodenogastric reflux [14]. Besides, diabetic microvascular lesion significantly reduces blood flow to gastric mucosa, causing gastric peristalsis to slow down as experts speculate that this may also be one of the reasons for gastroparesis [15].

It is demonstrated that people with gallbladder diseases are easier to have BRG [16, 17]. As a bile reservoir and concentrator, a functioning gallbladder roughly outputs only 20–25% of hepatic bile directly into the gut. In cases of cholecystolithiasis and cholecystic polyps, the amount of hepatic bile that passes into the duodenum increases owing to the declining rate of water absorption in the gallbladder [18]. As far as cholecystectomy is concerned, on the one side, total hepatic bile enters the duodenum, which can produce a continuous flow and exceed the clearing capacity of the duodenum [19]. Furthermore, the pressure of the bile duct relatively increases, leading to powerful discharge of bile from the bile duct to the duodenum, which is more likely to cause disorders of gastrointestinal peristalsis. On the other side, postoperative injury of the direct neural pathway between the gallbladder and duodenum may bring about sphincter of Oddi dysfunction [20], and as a result, duodenogastric reflux comes up. However, some scholars have also reported that cholecystectomy is not involved in the occurrence of bile reflux [21].

Psychological factors play a momentous role in a variety of gastrointestinal illnesses, including BRG [22–24]. Through person's mood activity as a media, which is so-called brain-gut axis, it affects the function of body's internal organs, especially the alimentary canal. Via the feedback of the brain-gut axis, abnormal emotional activities and stress can regulate the synthesis and release of gastrointestinal hormones, thus causing disorders of gastroduodenal coordinated movement [25]. In endoscopy, the distribution of erosion often appears longitudinal in anxious people while annular in depressed ones. From another point of view, uncomfortable symptoms that originate from bile reflux may bring about mental stress, which leads to a vicious circle. Exactly as Yang et al. [26] indicated, both the self-rating anxiety scale (SAS) and the self-rating depression scale (SDS) scores in patients with reflux were statistically higher than those in healthy individuals. Based on the above, patients may benefit more through psychosocial intervention combined with conventional drug therapy.

The relationship between bile reflux gastritis and *Helicobacter pylori* (*H. pylori*) is undefined and contentious. On the one hand, *H. pylori* infection can increase the secretion of gastrin that reduces peristalsis in the gastric antrum, which promotes the occurrence of BRG. On the other hand, it is considered that raised pH value on account of alkaline bile in the stomach, and impaired mucus-bicarbonate barrier, are able to affect and destroy the environment of survival and colonization for *H. pylori*, and meanwhile, a high concentration of bile acids can directly kill *H. pylori* [27]. As a consequence of these, the infection rate of *H. pylori* in patients with BRG decrease. On the contrary, some academics declared the prevalence of *H. pylori* in BRG patients was similar to or beyond that in normal persons [28]. Diverse conclusions reached in previous studies may be due to the small number of cases covered in these studies. Therefore, large-scale researches are urgently required to demonstrate the association between them. Additionally, whether BRG patients need eradication treatment of *H. pylori* puzzles clinicians. Agin and Kayar [29] recommended eradicating *H. pylori* on account of its damage to mucosa.

## 4. Carcinomatous Risk

More and more studies have established that bile reflux gastritis is closely associated with the development of precancerous lesions and gastric cancer (GC) and may be an independent risk factor for GC [11, 30, 31]. A multicenter, cross-sectional, and observational study conducted in five centers in China concluded that independent risk factors for cancerous gastric lesions were the grade of bile reflux, patient's age, dietary habits, and family history of GC [32]. Another study that included 28,745 cases confirmed that age, male gender, gastric ulcer, bile reflux, *H. pylori* infection, and severe degree of chronic and acute inflammation to be risk factors for intestinal metaplasia [33]. As Kondo [34] supposed, the recurrence of gastric stump cancer might be triggered by bile reflux. In addition, the concentration and duration of exposure to bile have a positive correlation with the incidence rate of GC.

The mechanism of GC induced by bile reflux is still unclear. Microscopically, it was reported that exposure to a high concentration of bile acids increased the production of reactive oxygen species (ROS) and reactive nitrogen species (RNS), which could cause DNA damage and mutations of genes like p53, participating in human carcinogenesis [35]. Besides, histologic changes of gastric mucosa caused by bile reflux could experience a procedure from intestinal metaplasia to dysplasia and even adenocarcinoma.

Because of the fluidity and gravity of bile juice, GC mostly occurred in the rear part and greater curvature of the stomach. In particular, physicians should pay more attention to these sites in patients with bile reflux in order to identify gastric tumor early.

## 5. Diagnosis

**Figure 2:**
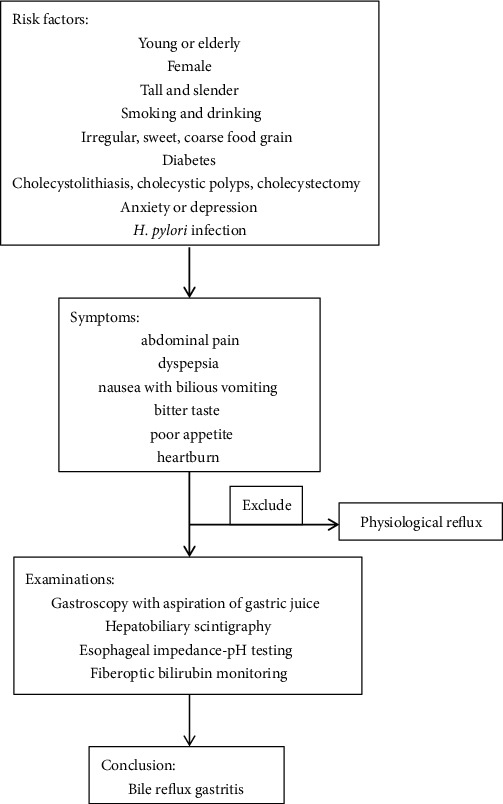
Flow chart for the diagnostic pathway.

The diagnosis of bile reflux gastritis is still challenging for its atypical clinical symptoms and nonspecific auxiliary examinations (Figure 2). Patients with BRG often complain of abdominal pain, dyspepsia, nausea with bilious vomiting, bitter taste, poor appetite, and heartburn while some patients even do not have symptoms. Additionally, the severity of these symptoms was not found to be proportional to the amount of bile in the reflux [36].

In prior studies, it was revealed that there might be a small amount of duodenogastric reflux physiologically in the stomach after feeding and in fasting [37]. Furthermore, the feeling of nausea during gastroscopy may be involved in a reverse flow of duodenal contents. Therefore, it brings trouble for clinicians in discriminating physiological reflux from pathological reflux. Thanks to the finding that levels of conjugated and unconjugated bile acids in the stomach were almost equal in normal individuals while conjugated bile acids increased in patients with BRG; clinicians can regard the ratio of conjugated bile acids to total bile acids as a criterion for distinction between physiological and pathological reflux [38].

Although there is no golden standard for diagnosis of BRG, four vital techniques including hepatobiliary scintigraphy, gastroscopy with aspiration of gastric juice, fiberoptic bilirubin monitoring, and esophageal impedance-pH testing are commonly recognized [39]. In summary, each of them has its own merits and demerits (Table 1).

First, hepatobiliary scintigraphy that shows radiotracer in the stomach to prove reflux is deemed as the least invasive investigation with good tolerability and sensitivity [40]. However, price and radiation exposure limit its application.

**Figure 3:**
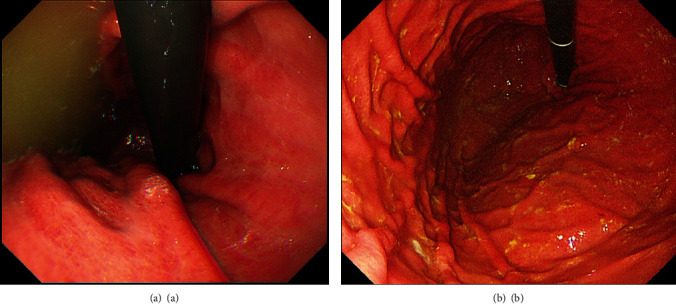
Endoscopic presentation of bile reflux gastric. (a): Bile lake; (b): bile stain.

Second, as one of the most widely used inspections in the digestive system, endoscopy can straightly evaluate the current status of the stomach. After insertion of gastroscope for over one minute, continuous gastrointestinal reflux can be seen in patients [41]. Compared with bile lake, which is unable to be confirmed using the scintigraphic method, visualization of bile stain more effectively indicates the retention of a large volume of bile juice in the stomach [42] (Figure 3). What's more, appearances of hyperemia, edema and erythema, which are frequently observed in patients with BRG, can assist endoscopic diagnosis. In other aspects, biopsy of gastric mucosa shows histologic features of atrophy and intestinal metaplasia. Nevertheless, lack of specificity in terms of endoscopic manifestations imposes restriction on its practical value. As for aspiration of gastric fluids, it enables chemical analysis of the composition of fluids to testify the presence of bile acids. However, the decreased detection rate owing to the periodicity of pathological reflux may limit its utility.

Third, Bilitec 2000, based on the theory that bilirubin absorbs light at a specific wavelength, can deduce the existence of bile as it is demonstrated in vitro studies that there is a statistically significant relevance between the concentration of bilirubin and bile acids, which suggests that bilirubin can be an alternative marker for bile reflux [43]. However, the technique is still immature because the measuring consequence can be easily influenced by various factors such as the pH and dilution of the refluxate.

Finally, esophageal impedance-pH testing, which is highly sensitive for all kinds of reflux, uses a combination of reflux data as a marker for the presence of bile reflux [39]. Although it is an advancement in the detection of reflux, it represents a measure of entire reflux instead of a particular measure of bile reflux.

## 6. Management

To date, there is still no official and unified therapeutic regimen for patients with BRG. Related studies on the treatment of BRG are ongoing, and as far as we know, the first priority is to eliminate risk factors. Patients ought to quit cigarettes and wine, control blood glucose, stick to a healthy and regular diet, keep cheerful and optimistic, and eradicate *H. pylori*.

Clinical trials are being conducted to validate and evaluate the therapeutic efficiency of various drugs. As a result, the use of ursodeoxycholic acid (UDCA), hydrotalcite, proton pump inhibitors (PPIs), and prokinetic agents are widely accepted for the treatment of BRG [44]. Besides, as bile adsorbents, cholestyramine has been found to be useful in treating patients with mild or moderate BRG in the past. UDCA, which has been proved to have explicit curative effects, plays a role both in protecting gastric mucosa and reducing reflux. On the one hand, UDCA can antagonize the cytotoxicity of hydrophobic bile acids, inhibit apoptosis, and clear free radicals to improve anti-oxidative ability. It was revealed that UDCA could facilitate the recovery of mucosa by lowering the level of the epidermal growth factor, which reflected the degree of damage in the gastric mucosa [45]. On the other hand, UDCA can also promote the excretion of endogenous bile acids, reduce bile viscosity, and accelerate the flow of bile. Furthermore, UDCA exists in the hepatoenteric circulation and still maintains a high concentration in gastric juice 14 days after withdrawal, which is beneficial in alleviating gastric mucosal inflammation and clinical symptoms [46]. Therefore, UDCA is recommended as the primary choice for BRG.

As for the other drugs mentioned above, hydrotalcite relieves abdominal discomfort to some extent by neutralizing bile acids and enhancing the effect of the mucosal barrier [8]. PPIs inhibit the secretion of gastric acids and can relieve digestive symptoms caused by acid reflux. Prokinetic agents aim to enhance gastric and duodenal peristalsis and accelerate gastric emptying. However, the efficacy of a single drug for secondary BRG is poor and the recurrence rate is high, which means that monotherapy is not capable of achieving the desired effect. Polytherapies such as UDCA combined with hydrotalcite were confirmed to be superior to other options [44].

Psychological interventions such as hypnotherapy, relaxation techniques, biofeedback, and cognitive behaviour therapy are likely to have a therapeutic value in patients with stress-related symptoms or reporting partial or complete lack of response to drug treatments. If none of the above works, surgical management of bile reflux with the purpose of diverting bile away from the stomach can be taken into consideration [47].

## 7. Conclusions

BRG is a conventional but poorly understood disease in clinical practice. The occurrence of BRG generally includes two parts. One is reflux that is considered to be linked to gastroduodenal dysmotility, disorder of gastroduodenal hormones, or surgeries. The other is inflammation, which mainly arises from the stimulation of bile acids and lysolecithin. Numerous factors are reported to be associated with BRG, including age, sex, body type, living habits, diabetes, gallbladder diseases, psychology, and *H. pylori* infection. More and more studies have demonstrated the carcinomatous risk of BRG. Hepatobiliary scintigraphy, gastroscopy, fiberoptic bilirubin monitoring, and esophageal impedance-pH testing are used for the diagnosis of BRG. With regard to treatment, the primary one is lifestyle modifications. UDCA combined with hydrotalcite is recommended compared with monotherapy. For those who fail drug therapy, psychological interventions and surgical management ought to be considered. Hopefully, this review will guide clinicians investigating BRG.

## Figures and Tables

**Table 1:** Comparison of diagnostic techniques for BRG.

Diagnostic techniques	Characteristics
Gastroscopy with aspiration of gastric juice	Widely used and accepted, convenient; poor specificity
Hepatobiliary scintigraphy	Sensitive, noninvasive, reproducible; expensive, radioactive, lack of anatomical resolution, unable to accurately quantify volume, concentration and the composition of the refluxate
Fiberoptic bilirubin monitoring	Reliable; poorly tolerated, immature
Esophageal impedance pH testing	Sensitive, reproducible, quantify acidic, weakly acidic and nonacidic reflux episodes; poor specificity, afflictive

## Data Availability

Figures of bile lake and bile stain come from digestive endoscopy center at the First Affiliated Hospital of Soochow University. The figure of pathogenesis and flow chart, as well as the table, are made by authors themselves.

## Abbreviations

BRG: Bile reflux gastritis
DGER: Duodenogastroesophageal reflux
GERD: Gastroesophageal reflux disease
*H. pylori*: *Helicobacter pylori*
GC: Gastric cancer
UDCA: Ursodeoxycholic acid.
